# Advances in iron-storage protein-based MRI reporter genes: from molecular mechanisms to translational imaging applications

**DOI:** 10.3389/fbioe.2026.1950409

**Published:** 2026-09-29

**Authors:** Haoxiang Li, Yucun Huang

**Affiliations:** Department of Radiology, Guangdong Provincial People’s Hospital, Zhuhai Hospital (Jinwan Central Hospital of Zhuhai), Zhuhai, Guangdong, China

**Keywords:** cell tracking, ferritin, gene therapy, iron-storage protein, molecular imaging, MRI reporter gene

## Abstract

Non-invasive monitoring of cellular and molecular events is essential for advancing precision medicine. While exogenous probes often suffer from signal dilution, iron-storage protein-based MRI reporter genes offer an alternative for longitudinal tracking, because contrast is regenerated by the cells themselves. Especially ferritin and its engineered variants have attracted growing interest due to their ability to regulate intracellular iron and generate endogenous T2/T2* contrast. This review summarizes the molecular mechanisms and design principles of iron-based MRI reporter genes, and examines recent developments including CRISPR-mediated integration and manganese-dependent “Bright Ferritin” platforms, which generate positive T1 contrast but require repeated administration of exogenous manganese. Preclinical studies have tracked cellular dynamics *in vivo* and monitored transgene expression after viral gene delivery, although the evidence base remains almost entirely preclinical. Nevertheless, a persistent challenge lies in generating sufficiently strong MRI contrast while maintaining the delicate balance of intracellular iron metabolism on which these reporters depend. By linking molecular design to imaging performance, this review distinguishes what has been demonstrated experimentally from what remains prospective, and identifies the quantitative and safety questions that stand between the current evidence and clinical use.

## 1 Introduction

Non-invasive monitoring of cellular and molecular events has become indispensable for precision medicine and targeted therapies. Traditional approaches relying on exogenous contrast agents frequently encounter signal dilution over time, limited retention, and inability to reflect dynamic biological processes (Cheng, 2023; He et al., 2015). These shortcomings have driven the development of genetically encoded magnetic resonance imaging (MRI) reporter genes that produce persistent, cell-autonomous contrast from within the target cells themselves.

Among the various MRI reporter systems, iron-storage protein-based reporters stand out for their unique combination of biocompatibility and endogenous contrast generation. Iron is an essential element in cellular physiology, tightly regulated through coordinated processes of metabolism. Ferritin, the primary intracellular iron-storage protein, can sequester thousands of iron atoms within its nanocage structure and thereby modulate local magnetic susceptibility (Theil, 1987). The mammalian ferritin heavy chain 1 (FTH1) and its engineered derivatives store iron within a protein nanocage as a ferrihydrite-like mineral, whereas the bacterial protein MagA supports biomineralization of a magnetite-like phase. Transferrin receptor-1 (TfR1) does not itself form a mineral core; it increases the quantity of iron available to these storage proteins by importing transferrin-bound iron (Gilad et al., 2008). Iron accumulated in this way increases local magnetic susceptibility and shortens T2 and T2* relaxation times, producing hypointense signal that is most conspicuous on gradient-echo sequences. For these T2/T2*-based reporters no exogenous agent is required, although this does not extend to the manganese-dependent variants discussed below.

Early studies established ferritin as a viable MRI reporter in small-animal models, yet the field has since evolved rapidly (Kim et al., 2013; Cohen et al., 2007). Recent years had witnessed substantial engineering refinements, including CRISPR/Cas9-mediated genomic integration for stable expression, promoter optimization for tissue-specific control, and the Bright Ferritin platform that enables on-demand signal recall for extended tracking of human embryonic and pluripotent stem cells (Son et al., 2024). These innovations have expanded the applicability of iron-storage reporters from basic cell labeling to real-time monitoring of transgene expression in AAV-mediated gene therapy and early detection of malignant transformation in transplanted cells (Naumova and Vande Velde, 2018). Despite these advances, dedicated syntheses focused exclusively on iron-storage protein-based MRI reporters remain scarce. Existing reviews either cover broad MRI reporter categories or emphasize other modalities such as CEST-based or optical systems (Brindle, 2022). The development of iron-storage MRI reporter genes in the past 2 decades is summarized in Figure 1.

**FIGURE 1:**
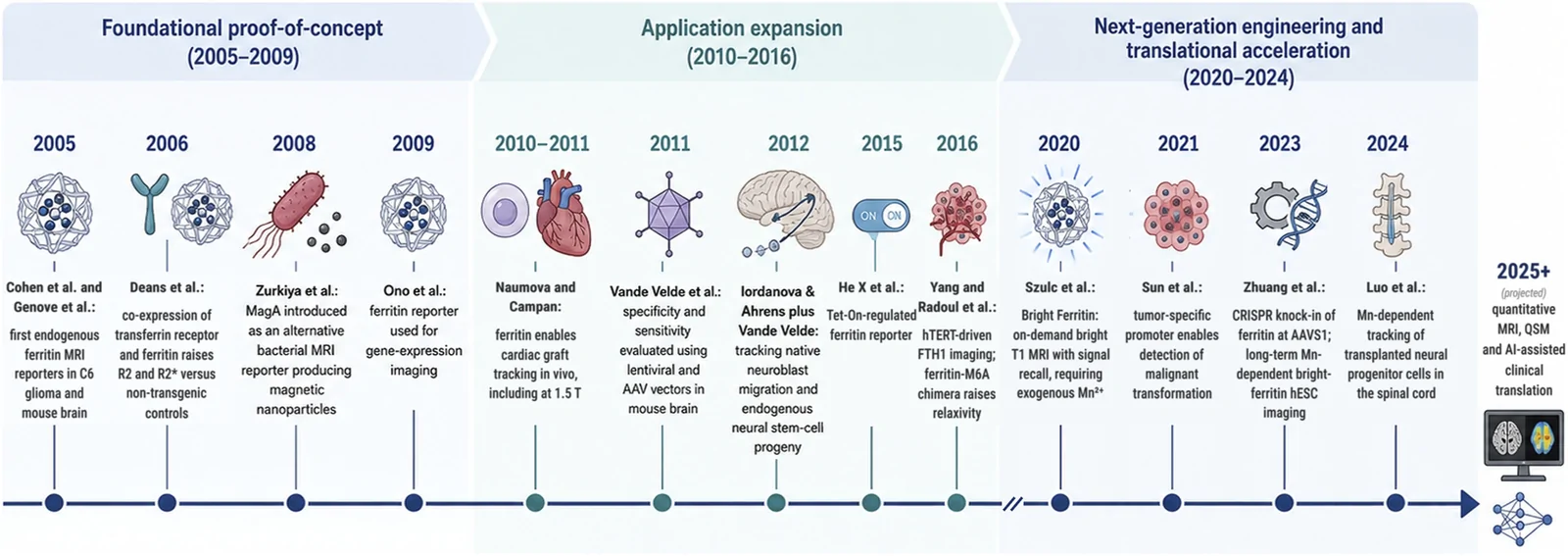
Evolution of iron-storage protein-based MRI reporter genes over the past 2 decades. Early investigations established ferritin as an endogenous MRI reporter capable of generating detectable T2/T2* contrast through intracellular iron sequestration. Subsequent developments expanded reporter performance through modulation of iron uptake pathways, incorporation of bacterial iron-biomineralization proteins such as MagA, and engineering of ferritin variants with enhanced magnetic properties. More recent advances include CRISPR/Cas9-mediated genomic integration, inducible and activity-responsive genetic circuits, and the emergence of Bright Ferritin systems, in which overexpressed wild-type ferritin sequesters exogenously administered manganese to give positive T1 contrast that can be recalled on demand for long-term monitoring of stem-cell fate and gene therapy. Current efforts are increasingly focused on quantitative imaging, multimodal integration, and clinical translation.

This review is organised around a single question: what determines whether an iron-storage reporter gene produces contrast that can actually be detected, and where does that chain of determinants break down? Detectability is set by three terms in series—the quantity of metal a cell can be made to accumulate, the relaxivity per metal atom conferred by the mineral phase that forms, and the fraction of the resulting relaxation difference that survives the voxel size, field strength and pulse sequence used to measure it. Each section addresses one link in this chain. Section 2 covers the molecular machinery that sets metal loading and the magnetic properties of the stored mineral, together with the quantitative relationships that govern detectability. Section 3 covers the genetic and protein engineering used to control expression and loading. Section 4 asks what has been demonstrated in animals. Section 5 examines where the chain fails. Section 6 places the platform alongside CEST and multimodal reporters, and Section 7 considers what clinical translation would require. Throughout, findings demonstrated experimentally are distinguished from applications that remain proposed.

This is a narrative rather than a systematic review. PubMed, Web of Science and Scopus were searched from database inception to March 2026, using combinations of the terms “MRI reporter gene”, “ferritin”, “FTH1”, “transferrin receptor”, “MagA”, “magnetoferritin”, “bright ferritin”, “cell tracking” and “magnetic resonance”, supplemented by hand-searching the reference lists of retrieved articles. English-language primary studies and reviews reporting MRI contrast attributable to a genetically encoded iron- or manganese-handling protein were considered; studies using exogenous nanoparticle labels alone, and conference abstracts without full-text data, were excluded. Where the literature is contradictory, the conflicting findings are reported rather than reconciled.

## 2 Molecular mechanisms and design principles of iron-storage MRI reporters

The development of genetically encoded MRI reporters is fundamentally rooted in the ability to reprogram native metal homeostasis. Unlike conventional exogenous contrast agents, which rely on passive biodistribution and cellular endocytosis, iron-storage reporters function as active, intracellular metabolic sinks. They assemble mineral cores *in situ* by sequestering iron from the host’s labile pool; depending on the protein employed, the resulting mineral ranges from weakly magnetic ferrihydrite to superparamagnetic magnetite, a distinction that has large quantitative consequences (Section 2.5). Over the past 2 decades, the design philosophy of these systems have undergone a profound evolution. This trajectory begins with the straightforward overexpression of native mammalian metalloproteins, expands into the synergistic modulation of iron transport networks and alternative bacterial biomineralization pathways, and ultimately culminates in the sophisticated structural engineering of next-generation chimeric variants. The unifying principle across all these strategies is the pursuit of maximizing localized magnetic susceptibility for robust radiological detection while rigorously preserving the overall metabolic integrity of the host cell.

### 2.1 Ferritin-based MRI reporter systems: molecular basis of contrast generation

Iron-storage protein-based MRI reporters harness the cell’s natural iron homeostasis machinery to generate contrast intrinsically. For the T2/T2* reporters considered in this section no exogenous agent is required; the manganese-dependent variants described in Sections 2.4, 5.2 are an explicit exception. At the core of this system lies ferritin, a ubiquitous 24-subunit nanocage assembled from heavy and light chains. The heavy chain subunit (FTH1) possesses ferroxidase activity that rapidly oxidizes ferrous iron (Fe^2+^) to ferric iron (Fe^3+^), facilitating its safe sequestration within the protein shell as a ferrihydrite-like mineral core. The magnetic state of this core is frequently misdescribed and is worth stating precisely. Native ferritin is not superparamagnetic: the ferrihydrite core is antiferromagnetically ordered, its small net moment arises from uncompensated surface spins, and its magnetisation does not saturate at accessible field strengths (Sudarev et al., 2023). Superparamagnetic behaviour belongs instead to magnetite (Fe_3_O_4_) cores, whether biomineralized within apoferritin cages *in vitro* to give magnetoferritin or formed through bacterial pathways such as MagA. Perturbation of the local field by either mineral shortens T2 and T2*, producing hypointense contrast that is most conspicuous on gradient-echo sequences, but the efficiency with which the two minerals do so differs by more than two orders of magnitude per iron atom (Section 2.5). Importantly, ferritin-based reporters differ fundamentally from exogenous superparamagnetic iron oxide (SPIO) labeling. While SPIO particles provide strong initial contrast, their signals are diluted with cell division and may persist after cell death, leading to potential misinterpretation. In contrast, ferritin expression is genetically encoded and therefore maintained across cell generations, enabling longitudinal and cell-specific imaging.

The mammalian FTH1 serves as the prototype for most iron-storage MRI reporters. Overexpression of FTH1 increases intracellular iron uptake, often through coordinated upregulation of transferrin receptor-1 (TfR1), which imports transferrin-bound iron. The accumulated iron is then oxidized and stored, leading to measurable increases in transverse relaxivity (R2 and R2*) (Yang et al., 2023; Singhania et al., 2024) (Figure 2).

**FIGURE 2:**
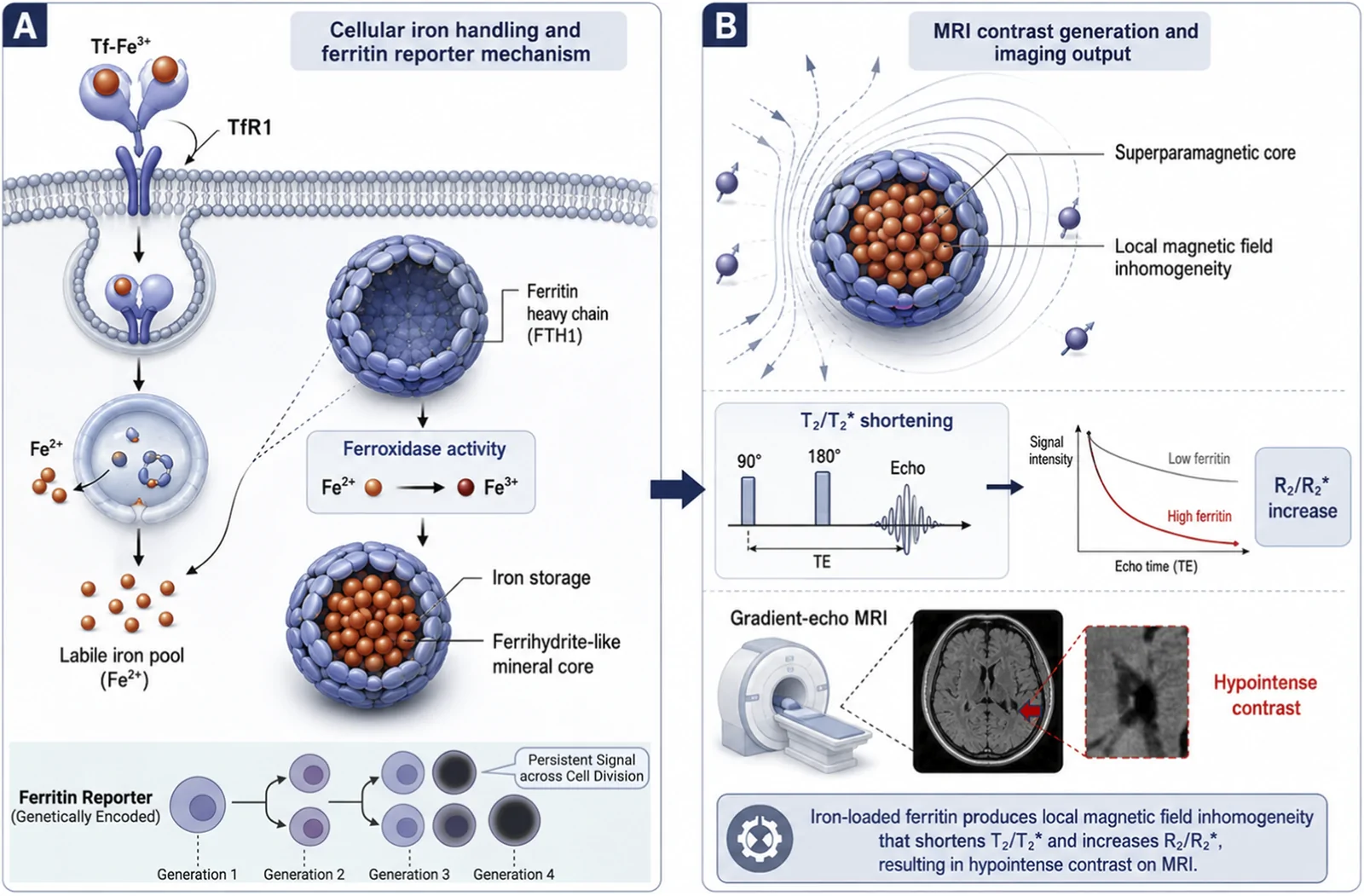
Molecular basis of MRI signal generation mediated by ferritin reporter. **(A)** Transferrin-bound iron is internalized through transferrin receptor-1 (TfR1)-mediated endocytosis and released into the intracellular labile iron pool. Ferritin heavy chain (FTH1) oxidizes ferrous iron (Fe^2+^) to ferric iron (Fe^3+^), promoting the formation of ferrihydrite-like mineral cores within the ferritin nanocage. **(B)** Progressive iron accumulation increases local magnetic susceptibility and accelerates transverse relaxation, resulting in shortening of T2 and T2* relaxation times and the generation of hypointense MRI signals. Reporter contrast can be enhanced through coordinated regulation of iron uptake, iron supplementation, or engineered ferritin variants while remaining dependent on cellular iron homeostasis.

### 2.2 Modulation of iron uptake: transferrin receptor-1 and synergistic strategies

Enhancing intracellular iron loading represents a key strategy for improving the sensitivity of ferritin-based MRI reporters. As mentioned above, TfR1 is a membrane protein responsible for iron uptake via transferrin-bound iron internalization and frequently co-expressed with ferritin to boost iron influx. TfR1-mediated endocytosis increases intracellular iron availability, which can subsequently be stored in ferritin. The evidence that this translates into greater MRI contrast is mixed, and it is worth setting out rather than summarising. Co-expression of human TfR1 and ferritin H-chain in a neural stem cell line raised R2 and R2* at both 1.5 T and 7 T relative to non-transgenic controls, and increased T2*-weighted contrast after transplantation into mouse brain (Deans et al., 2006). A systematic comparison of iron-handling reporter combinations similarly identified TfR1 together with DMT1 and a ferritin-M6A chimera as the most effective combination for T1-weighted contrast, although ferritin-M6A alone performed best on T2-weighted images (Hayashi et al., 2024). Set against this, overexpression of TfR1 in CHO-K1 cells raised intracellular iron but conferred no MRI contrast advantage over simply culturing non-transgenic cells in ferric citrate, leading those authors to conclude that TfR1 is not an effective reporter gene (Pereira et al., 2016); and in mesenchymal stem cells neither reporter alone significantly increased intracellular iron, with a small advantage from co-expression seen only at high extracellular iron concentrations (Pereira et al., 2015). A further qualification applies to the earlier engineered transferrin receptor literature, in which contrast was generated by an administered transferrin-conjugated iron oxide probe rather than by endogenous iron (Moore et al., 2001); those studies demonstrate receptor-mediated uptake of an exogenous agent and do not bear on endogenous-iron contrast. The balance of evidence is therefore that modulating iron uptake can raise reporter contrast, but that the effect is smaller and more context-dependent than is commonly stated, and in several systems is matched by iron supplementation alone. In addition to genetic modulation, iron supplementation strategies—such as ferric citrate and transferrin administration—have been employed to further increase intracellular iron pools and boost contrast generation. However, these approaches require careful optimization to avoid iron overload and oxidative stress, which can compromise cell viability and confound imaging results.

Together, these strategies converge on a core principle in reporter design: MRI contrast depends not simply on how strongly the reporter is expressed, but on how well the cell can supply iron and sustain the metabolic conditions that the reporter requires. This constraint recurs throughout the field: the supply of iron, rather than the abundance of the storage protein, is frequently the limiting term.

### 2.3 Magnetosome-associated proteins and alternative iron biomineralization strategies

Beyond mammalian ferritin, bacterial iron-handling systems provide alternative strategies for MRI reporter development. One notable example is MagA, a gene derived from magnetotactic bacteria that is involved in magnetosome formation and offers a complementary mechanism. It promotes the biomineralization of highly crystalline magnetite (Fe_3_O_4_) nanoparticles rather than ferrihydrite, yielding stronger superparamagnetic effects and higher R2 relaxivity in some contexts. When expressed in mammalian cells, MagA can form membrane-enclosed magnetosome-like structures that amplify MRI contrast while interacting with endogenous iron regulatory pathways (Zurkiya et al., 2008). Mechanistically, MagA facilitates iron transport and accumulation, promoting biomineralization processes distinct from ferritin-based storage. While ferritin forms ferrihydrite cores within a protein shell, MagA-associated pathways may lead to more crystalline or magnetite-like structures, potentially enhancing magnetic susceptibility effects. Whether this promise carries over into living mammalian cells is, however, a more complicated question. Several subsequent efforts found that the contrast enhancement MagA produces varies considerably with cell type and experimental conditions, making consistent results difficult to achieve—a problem that probably reflects how sensitive bacterial iron biomineralization is to the metabolic milieu of a host cell. The associated accumulation of iron in a biologically unfamiliar form can also exact a cost to cell viability, one that tends to become more apparent only as experiments extend over longer timeframes (Shen et al., 2018).

These findings highlight a broader design principle: effective MRI reporter genes must balance contrast efficiency with cellular compatibility. Although bacterial systems can offer superior magnetic performance, successful deployment in mammalian cells ultimately depends on how well these reporters can be accommodated by the host cellular machinery.

### 2.4 Engineered ferritin variants and next-generation reporter platforms

Recent advances in protein engineering have enabled the development of next-generation iron-storage MRI reporters with improved functionality. The Bright Ferritin platform is the most developed of these, and its mechanism is often described imprecisely. The ferritin protein itself is not mutated: cells are engineered to overexpress wild-type human ferritin, and the change in contrast mechanism arises from the metal that is supplied rather than from a change in the cage. When manganese is made available, the excess apoferritin sequesters Mn^2+^ into a discrete mineral core rather than leaving it in ionic form, and the resulting manganese-loaded ferritin acts as a T1-shortening agent with a reported relaxivity of 17.7 mM^-1^s^-1^, lowering cellular T1 by approximately 2.5-fold relative to wild-type cells (Szulc et al., 2020). The readout is therefore positive rather than hypointense and, because it depends on the metal supplied rather than on a label accumulated at the outset, it can be extinguished and recalled by withholding and re-administering manganese. This is the platform’s principal advantage and, equally, its principal constraint: contrast exists only after manganese administration, so Bright Ferritin is not probe-free in the sense that applies to the T2/T2* iron reporters, and the associated dose considerations are addressed in Section 5.2. Subsequent studies have used the platform to track human embryonic stem cells and neural progenitor cells over weeks to months (Zhuang et al., 2023).

Two further mechanistic levers are available. The first is spatial. Because transverse relaxation depends on how susceptibility is distributed in space and not on total iron content alone, the same quantity of iron produces a larger effect when it is concentrated into fewer, larger deposits than when it is dispersed; redirecting ferritin to a subcellular compartment exploits this by clustering iron and shifting the system toward the static dephasing regime described in Section 2.5 (Iordanova et al., 2013). The second is compositional: altering the ferritin sequence changes the mineral that forms within the cage, and therefore the relaxivity per iron atom, independently of how much iron is loaded (Radoul et al., 2016). The engineering methods used to realise these two principles, and the evidence supporting them, are considered in Section 3.2.

A unifying feature of these engineered reporters is their ability to harness endogenous iron metabolism and convert it into a sustained imaging signal. Reporter expression promotes the formation of intracellular iron-rich complexes that remain detectable over extended periods, allowing MRI contrast to be generated from within the cell rather than introduced externally. This property distinguishes iron-storage reporters from superparamagnetic iron oxide nanoparticle labeling, where signal intensity gradually diminishes as cells divide and the label becomes diluted. Because contrast is continuously replenished through reporter gene expression, engineered iron-storage systems are particularly well suited for monitoring cell fate over prolonged time scales (Table 1).

**TABLE 1:** Representative iron-storage protein-based MRI reporter systems and their design characteristics. FTH1, ferritin heavy chain 1; TfR1, transferrin receptor-1; FtMt, mitochondrial ferritin; MRI, magnetic resonance imaging.

Reporter system	Molecular mechanism	Source	MRI contrast mechanism	Major advantages	Main limitations	Representative references
Ferritin Heavy Chain (FTH1)	Ferroxidase-mediated oxidation of Fe^2+^ and storage as ferrihydrite within ferritin nanocages	Mammalian	Increased intracellular iron accumulation shortens T2/T2* relaxation times	Excellent biocompatibility; genetically encoded; stable through cell division	Moderate sensitivity; contrast depends on endogenous iron availability	(Cohen et al., 2005); (Yang et al., 2023; Singhania et al., 2024)
Ferritin + TfR1 Co-expression	Enhanced transferrin-mediated iron uptake coupled with ferritin storage	Mammalian	Increased intracellular iron loading amplifies susceptibility effects	May increase iron accumulation; evidence for improved contrast relative to ferritin alone is mixed	Potential disturbance of iron homeostasis; risk of oxidative stress; benefit not reproduced in all cell types, and comparable contrast is achievable by iron supplementation alone	(Deans et al., 2006); (Pereira et al., 2015); (Pereira et al., 2016)
MagA	Magnetosome-associated iron transport and biomineralization	Magnetotactic bacteria	Formation of magnetite-like particles producing strong susceptibility effects	Stronger magnetic contrast potential	Variable performance in mammalian cells; possible cytotoxicity and poor reproducibility	(Zurkiya et al., 2008); (Shen et al., 2018)
Mitochondrial Ferritin (FtMt)	Subcellular redistribution of iron storage to mitochondria	Mammalian	Localized iron accumulation alters relaxation properties	Enables compartment-specific imaging; tunable contrast	More complex biology; lower general applicability	(Iordanova et al., 2013)
Ferritin-M6A Chimera and Humanized Ferritin Variants	Protein engineering to improve iron biomineralization and expression efficiency	Engineered mammalian ferritin	Enhanced iron loading increases relaxivity	Improved expression stability; reduced immunogenicity; enhanced MRI sensitivity	Requires extensive validation for different cell types	(Radoul et al., 2016)
Bright Ferritin	Overexpressed wild-type human ferritin sequesters exogenously supplied Mn^2+^ into a mineral core	Mammalian (wild-type ferritin; engineered cells)	Mn-loaded ferritin shortens T1, producing positive contrast that can be recalled on demand	Longitudinal cell tracking; reduced baseline signal; compatible with stem-cell imaging	Requires repeated systemic MnCl_2_ administration at each imaging session; relatively limited *in vivo* validation	(Szulc et al., 2020); (Zhuang et al., 2023)

### 2.5 Quantitative basis of reporter-derived contrast: relaxivity, field dependence and detectability

Discussions of iron-storage reporters frequently quote relaxivity values without stating the basis on which they are normalised, and the resulting figures are not comparable. Transverse relaxivity may be expressed per millimolar iron, per millimolar ferritin cage, or per unit mass of iron per gram of tissue; because a single cage may hold of the order of 10^3^ iron atoms, these conventions differ by approximately three orders of magnitude. The same magnetoferritin preparation has been reported as 78 mM^-1^s^-1^ on a per-iron basis and 4.04 × 10^5^ mM^-1^s^-1^ on a per-particle basis (Clavijo Jordan et al., 2010). Values quoted below are per millimolar iron unless stated otherwise.

On that basis, the difference between the two mineral phases is large and is not explained by iron content. At 7 T, native ferritin with a loading factor of 868 iron atoms per cage has r2 = 1.27 ± 0.23 mM^-1^s^-1^, whereas magnetoferritin at a comparable loading factor of 873 has r2 = 402 ± 72 mM^-1^s^-1^ — a difference of more than two orders of magnitude at matched iron content (Strbak et al., 2021). Longitudinal relaxivities differ far less (0.40 ± 0.07 against 1.21 ± 0.22 mM^-1^s^-1^), which is why these systems are read out on T2-and T2*-weighted rather than T1-weighted images. For the manganese-loaded ferritin of the Bright Ferritin platform the relevant figure is a longitudinal relaxivity of 17.7 mM^-1^s^-1^, an order of magnitude above that of either iron mineral, and this is what makes a positive T1 readout feasible (Szulc et al., 2020). The practical implication is that how much protein is expressed is a weaker lever on contrast than what mineral forms inside it.

Field strength enters differently for the two minerals, and this is the principal reason preclinical results do not transfer to clinical scanners. The transverse relaxation rate of ferritin solutions increases approximately linearly with B_0_ from 0.05 T to about 11.7 T, and the slope per unit iron is largely independent of loading factor (Vymazal et al., 1996; Gossuin et al., 2000). A weakly magnetic, unsaturated antiferromagnetic core behaves in this way because its own magnetisation rises with the applied field. This is not the behaviour predicted by outer-sphere relaxation theory, which requires a quadratic field dependence and additionally underestimates the observed rates; the discrepancy has been attributed to exchange of protons with the hydrated core surface (Gossuin et al., 2000). Superparamagnetic magnetite behaves differently again, since its moment saturates at modest field strengths. In consequence, contrast obtained from a ferritin reporter at 7 or 9.4 T in a rodent will be approximately two-to six-fold weaker at the 1.5–3 T available clinically, before any allowance is made for the larger voxels and shorter acquisitions of clinical protocols. Reported sensitivities are therefore uninterpretable unless the field strength is stated, a point on which the standardisation argument of Section 7 depends.

The final term is how much of the relaxation difference survives the pulse sequence. Transverse decay measured with a gradient echo, R2*, is conventionally separated into an irreversible component R2 and a reversible component R2′. A 180° refocusing pulse inverts accumulated phase, so any field offset that is constant over the echo time is exactly reversed and contributes to R2′ but not to R2; a gradient echo, having no such inversion, retains both. What renders an offset irreversible is molecular motion: a water molecule that diffuses far enough during the echo time to sample a different local field acquires phase before and after the refocusing pulse that no longer cancels. Whether a given deposit falls into the static-dephasing regime, in which diffusion is negligible and R2′ is large, or the motional-averaging regime, in which diffusion averages the perturbation and R2 approaches R2*, is set by the product of the induced frequency shift and the diffusion correlation time across the deposit (Yablonskiy and Haacke, 1994; Gillis and Koenig, 1987). Superparamagnetic deposits—magnetoferritin, MagA-derived magnetite, or exogenous iron oxide—produce large frequency shifts and lie close to the static-dephasing limit, which is why gradient-echo imaging is markedly more sensitive to them than spin-echo imaging. Native ferritin behaves differently: its transverse relaxation rate is essentially independent of interecho spacing (Gossuin et al., 2000), so the contrast it generates is largely irreversible and is not recovered by refocusing. This asymmetry is easily overlooked when sequences optimised for iron oxide labelling are transferred unchanged to ferritin reporters.

Combining these terms gives a quantitative account of the sensitivity gap between genetically encoded and exogenously labelled cells. Reporter gene expression in mesenchymal stem cells raised intracellular iron to approximately 0.3–1 pg per cell even with iron supplementation, whereas iron oxide labelling of the same cells routinely exceeds 5 pg per cell (Pereira et al., 2015) and clinical dendritic cell labelling has used approximately 30 pg per cell (Bulte, 2009). A ten-to hundred-fold deficit in iron per cell, multiplied by a two-to three-hundred-fold deficit in relaxivity per iron atom, is by itself sufficient to account for the difference in detectable cell number discussed in Section 5.1, without invoking any additional factor. Representative quantitative parameters are collected in Table 2.

**TABLE 2:** Quantitative reference values for iron- and manganese-storage MRI reporters. Relaxivity values are reported together with the basis on which they were normalised, because per-iron, per-cage and per-mass conventions differ by up to three orders of magnitude and are frequently conflated. Detection limits are reported together with the field strength, voxel dimensions and labelling method to which they apply. r1, longitudinal relaxivity; r2, transverse relaxivity; LF, loading factor (iron atoms per ferritin cage); s.c., subcutaneous.

Parameter	Reported value	Conditions and normalisation basis	Source
r2, native ferritin	1.27 ± 0.23 mM^-1^s^-1^	7 T; per mM Fe; LF 868	(Strbak et al., 2021)
r2, magnetoferritin	320–528 mM^-1^s^-1^	7 T; per mM Fe; LF 553–873	(Strbak et al., 2021)
r1, native ferritin	0.40 ± 0.07 mM^-1^s^-1^	7 T; per mM Fe; LF 868	(Strbak et al., 2021)
r1, magnetoferritin	1.21–2.17 mM^-1^s^-1^	7 T; per mM Fe	(Strbak et al., 2021)
Effect of normalisation basis	78 mM^-1^s^-1^ (per mM Fe) versus 4.04 × 10^5^ mM^-1^s^-1^ (per mM particle)	Same magnetoferritin preparation; the two conventions differ by a factor of approximately ×5 10^3^	(Clavijo Jordan et al., 2010)
r1, Mn-loaded ferritin (Bright Ferritin)	17.7 mM^-1^s^-1^	Cellular T1 reduced approximately 2.5-fold relative to wild-type cells	(Szulc et al., 2020)
Field dependence of ferritin R2	Approximately linear in B_0_ over 0.05–11.7 T; slope per unit iron independent of LF	Ferritin solutions; contrast at 7–9.4 T is approximately two- to six-fold greater than at 1.5–3 T	(Vymazal et al., 1996; Gossuin et al., 2000)
Echo-time dependence, native ferritin	T2 essentially independent of interecho spacing	Contrast largely irreversible; not recovered by spin-echo refocusing	(Gossuin et al., 2000)
Iron per cell, reporter gene overexpression	≈0.3–1 pg Fe per cell	Mesenchymal stem cells; FTH1 and/or TfR1, with iron supplementation	(Pereira et al., 2016)
Iron per cell, exogenous iron oxide labelling	>5 pg Fe per cell (*in vitro*); ≈30 pg Fe per cell (clinical dendritic cells)	Same cell line *in vitro*; ferumoxides clinically	(Pereira et al., 2015); (Bulte, 2009)
Clinical cellular MRI sensitivity	≈1.5 × 10^4^ cells	3 T; 0.5 × 0.5 × 3.5 mm voxel; ferumoxide-labelled dendritic cells; cross-calibrated against^111^In-oxine	(Bulte, 2009)
Single-cell detection, exogenous label	Single labelled cells detected *in vivo*	Micron-sized iron oxide particles; high-resolution gradient-echo imaging	(Shapiro et al., 2006)
MnCl_2_ dose, Bright Ferritin *in vivo*	0.4 mmol/kg (mouse); 0.2 mmol/kg (rat), s.c., repeated per session	Compare 5 μmol/kg for clinical mangafodipir	(Zhuang et al., 2023); (Luo et al., 2024)

## 3 Recent advances in genetic engineering and optimization

The past 5 years have transformed iron-storage MRI reporters from simple overexpression constructs into precision-engineered genetic systems that are increasingly ready for clinical pipelines. Rather than merely increasing ferritin levels, contemporary efforts have focused on three closely related objectives: (i) stable genomic integration, (ii) precise regulatory control, and (iii) context-dependent functional responsiveness, while maintaining an acceptable safety profile. Together, these developments have expanded the utility of iron-storage reporters beyond experimental labeling systems, enabling their incorporation into emerging gene- and cell-based therapeutic platforms.

### 3.1 Transcriptional control and genetic circuit engineering

A transformative breakthrough is the engineering of context-dependent transcriptional control, where reporter gene expression is strictly governed by native or rationally designed promoters. This genetic wiring ensures that MRI signals remain completely dormant until triggered by a precise microenvironmental cue. Rather than yielding a static anatomical map, the contrast agent can be programmed to exclusively illuminate the oxygen-starved core of a tissue. In other applications, the probe selectively maps the trajectory of a localized inflammatory response, or independently unmasks the subtle onset of malignant cellular transformation. For example, tumor-responsive promoters (e.g., PEG3) with translational enhancers (bFGF-2 5′UTR) and mRNA stabilizers (WPRE) have been used to drive ferritin heavy chain (FTH1) expression, enabling selective visualization of tumorigenic processes while minimizing background signal from non-pathological cells (Hu et al., 2025). Ultimately, such dynamic regulatory designs offer a profound upgrade in biological specificity, actively suppressing the false-positive background inherently associated with conventional constitutive reporters.

Building upon spatially restricted designs, modern reporting strategies increasingly incorporate synthetic gene circuits to achieve temporal precision. To achieve this, researchers are moving beyond rudimentary promoter designs to engineer sophisticated synthetic circuits and inducible platforms. This transition grants investigators the power to toggle reporter activity strictly on demand, thereby sparing host cells from the prolonged metabolic burden of continuous contrast agent production. Historically, deploying MRI reporters relied on viral vectors that blindly scattered foreign DNA across the host genome. Such unpredictable integration routinely triggered the silencing of the artificial cassette over time, while simultaneously risking the dangerous disruption of essential native genes. Targeted integration with CRISPR/Cas9 addresses part of this problem. Rather than relying on random insertion, FTH1/FTL1 cassettes can be knocked into candidate safe-harbour loci such as AAVS1, and human pluripotent stem cells modified in this way have maintained ferritin expression and MRI contrast through extended expansion and differentiation, with no reported loss of pluripotency (Hou et al., 2026; Zhuang et al., 2026). It should not be inferred from this that site-specific integration eliminates genotoxic risk. Cas9-induced double-strand breaks can generate large on-target deletions, chromosomal translocations, megabase-scale loss of heterozygosity and chromothripsis in addition to off-target editing, and the editing process itself selects against cells with an intact p53 damage response (Aussel et al., 2025). The designation of AAVS1 as a safe harbour is provisional rather than established: in human induced pluripotent stem cells, transgenes knocked into this locus showed highly variable expression between clones and were silenced during cardiomyocyte differentiation (Bhagwan et al., 2020). Targeted integration therefore reduces, but does not remove, the requirement for clonal characterisation and long-term genomic surveillance. These cells maintain consistent MRI contrast over months *in vivo*, providing a reliable foundation for long term cell tracking studies. As a result, reporter activity can be coupled more closely to cellular function, allowing MRI readouts to provide information beyond simple cell localization.

### 3.2 Protein engineering and subcellular optimization of reporter systems

Parallel to transcriptional regulation, protein-level engineering has emerged as a central strategy for enhancing MRI reporter performance, particularly in iron-based systems such as ferritin. The Bright Ferritin platform, whose mechanism is described in Section 2.4, is implemented at the genetic rather than the protein level: wild-type human ferritin is placed under a constitutive or inducible promoter, in the most recent work by CRISPR-mediated knock-in at AAVS1, and manganese is supplied exogenously when imaging is required (Szulc et al., 2020; Zhuang et al., 2026). Reported *in vivo* regimens are 0.4 mmol/kg MnCl_2_ given subcutaneously in mice and 0.2 mmol/kg in rats, administered 24 h before imaging and repeated at each subsequent session (Zhuang et al., 2023; Luo et al., 2024). Whether the resulting T1 contrast is more sensitive than T2* darkening depends on the tissue and the sequence and has not been settled by a controlled comparison; the advantage demonstrated so far is that a positive signal can be switched on at a chosen time point, which separates viable reporter-expressing cells from static susceptibility artefacts.

Subcellular targeting is achieved in practice by fusing mitochondrial presequences to ferritin subunits, or by deleting the mitochondrial targeting signal of FtMt to promote cytoplasmic accumulation. Increases in R2* were obtained without measurable disruption of overall cellular iron homeostasis, although these comparisons were made in cultured cells over short intervals and have not been repeated *in vivo* (Zeng et al., 2025). These observations suggest that intracellular localization is an important determinant of MRI reporter behavior.

In addition, to address immunogenicity concerns that previously hindered clinical translation, humanized ferritin variants have been developed. These strategies are particularly relevant for translational applications, where long term safety and reproducibility are critical. Humanized ferritin nanocages have been reported to express stably in primary human cells, and their use is motivated by an expectation of lower immunogenicity than non-human or bacterial constructs (Gruberova et al., 2025; Barolo et al., 2024). That expectation has not, however, been tested directly for a ferritin reporter in an immunocompetent host over a clinically relevant interval, and the immunogenicity of the transgene product, as distinct from that of the delivery vector, remains an open question (Section 5.3). Recent advances in protein engineering have improved reporter performance without imposing a substantial burden on cellular physiology, an important consideration for future clinical translation.

### 3.3 Functional and activity-based reporter design

Beyond improving signal intensity, a key emerging direction is the development of functional MRI reporters that encode dynamic biological processes. By linking reporter gene expression to differentiation programs or signaling pathways, MRI can be used to monitor cell fate transitions and functional states *in vivo*. For example, FTH1-based reporters driven by lineage-specific regulatory elements have been used to track neuronal differentiation of stem cells, demonstrating that MRI signals can reflect not only cell localization but also cellular phenotypic evolution (He et al., 2023).

A further possibility, so far largely unrealised, is to couple reporter expression to the microenvironment rather than to lineage. Because ferritin lies at the intersection of iron handling, oxidative stress and ferroptotic cell death (Ru et al., 2024), a cassette placed under a hypoxia- or ferroptosis-responsive element would in principle report metabolic state rather than cell position. We are not aware of a study that has demonstrated such a biosensor with an iron-storage reporter *in vivo*, and the designs incorporating feedback regulation and tunable expression that are discussed in this context remain proposals rather than results. The distinction matters, because a reporter whose output depends on the same iron pool it is intended to sense introduces a circularity that will have to be resolved experimentally before such readouts can be interpreted. Such designs move MRI reporter genes beyond passive labeling and toward systems that can report biologically meaningful changes as they occur in living tissues.

The advances described above have substantially expanded the capabilities of iron-storage MRI reporters. Although early studies focused primarily on enhancing cellular MRI contrast, subsequent work has expanded the role of ferritin reporters beyond simple cell labeling, opening new opportunities for monitoring biological processes *in vivo*. Continued refinement of these technologies is expected to facilitate their integration into future cell-based therapies and translational imaging studies (Table 3).

**TABLE 3:** Recent advances in the genetic engineering and optimization of iron-storage MRI reporters.

Engineering category	Specific strategy	Key modification	Major benefit	References
Transcriptional Control & Genetic Circuits	Context-Dependent Promoters	Tumor-responsive PEG3 promoter coupled with bFGF-2 5′UTR and WPRE elements	Eliminates constitutive background; restricts MRI contrast strictly to malignant transformation events	(Hu et al., 2025)
​	CRISPR/Cas9 Genomic Integration	Site-specific knock-in of FTH1/FTL1 cassettes into AAVS1 safe-harbor loci	Reduces, but does not eliminate, insertional mutagenesis and silencing; pluripotency preserved in the studies reported to date	(Hou et al., 2026; Zhuang et al., 2026)
Protein Engineering & Subcellular Optimization	“Bright Ferritin” Platform	Overexpression of wild-type human ferritin, which sequesters exogenously administered Mn^2+^; expression may be constitutive or inducible	Shifts readout to positive T1 contrast and allows on-demand signal activation, distinguishing viable grafts from static artefacts; requires systemic MnCl_2_ at each imaging session	(Szulc et al., 2020); (Zhuang et al., 2026)
​	Subcellular Targeting	Addition of mitochondrial presequences or deletion of targeting peptides to spatially confine ferritin	Increases R2* by concentrating iron pools; demonstrated in cultured cells over short intervals	(Zeng et al., 2025)
​	Humanized & Codon-Optimized Variants	Deployment of fully humanized chimeric nanocages and codon-optimized FTH1 transcripts	Intended to reduce immunogenicity and stabilise expression; not yet tested for a ferritin reporter in an immunocompetent host over a clinically relevant interval	(Gruberova et al., 2025; Barolo et al., 2024)
Functional & Activity-Based Reporters	Lineage-Specific Reporters	FTH1 transcription stringently regulated by lineage-committed promoters (e.g., NSE)	Translates anatomical tracking into functional mapping of *in vivo* cellular differentiation and neurogenesis	(He et al., 2023)
​	Microenvironment Biosensors	Integration of feedback-regulated circuits responsive to distinct triggers (e.g., hypoxia, ferroptosis-induced iron flux)	Proposed as a dynamic *in vivo* biosensor of metabolic state; no *in vivo* demonstration with an iron-storage reporter has been reported to date	(Ru et al., 2024)

## 4 Preclinical applications

The story of iron-storage MRI reporters in living animals began in the mid-2000s with the first demonstrations that genetically encoded ferritin could produce detectable contrast without any injected agents. Building on these foundational concepts, subsequent preclinical investigations have systematically unlocked the true diagnostic power of ferritin reporters. Rather than simply marking an anatomical location, these genetically encoded probes have evolved into comprehensive surveillance tools. From the first brain and tumor models to today’s sophisticated cardiac and liver applications, the field has progressed to longitudinal imaging strategies capable of interrogating biological processes *in vivo*.

### 4.1 Tracking of cellular processes and disease mechanisms

Transitioning from molecular design to *in vivo* application, iron-storage reporters have become indispensable tools for visualizing dynamic cellular networks. The ability to harness endogenous iron for continuous contrast generation allows researchers to bypass the temporal limitations of traditional exogenous labeling.

#### 4.1.1 Monitoring cell fate and differentiation

The first *in vivo* demonstrations came in 2005, when ferritin overexpression in rodent brain produced clear hypointense signals on T2*-weighted images, proving that iron-storage reporters could label cells non-invasively (Genove et al., 2005). Shortly afterward, the same approach was used to track glioma cells and visualize their growth *in situ* (Cohen et al., 2005). Another study demonstrated that neural stem cells engineered to express ferritin, which is often in combination with transferrin receptor, could be detected *in vivo* following transplantation into the brain, with MRI signals reflecting both cell localization and proliferation (Deans et al., 2006). In these studies, reporter-associated contrast provided information on cell localization and, when supported by histological validation, changes in graft persistence or expansion. As the field matured, the focus shifted toward the prolonged monitoring of regenerative therapies. For instance, mesenchymal stem cells (MSCs) engineered with FTH1 and TfR1 generated sustained contrast in musculoskeletal and spinal cord injury models, permitting accurate assessments of long term graft retention (Pereira et al., 2015). Subsequent studies extended these observations by linking ferritin expression to lineage-specific promoters. NSE promoter-driven FTH1 enabled direct visualization of neuronal differentiation of bone-marrow-derived mesenchymal stem cells in rat spinal cord injury, with progressive hypointense signals correlating tightly with NeuN-positive neuron formation over 8 weeks (He et al., 2023). Bright Ferritin-labeled human neural stem cells have similarly demonstrated long term survival and directional migration in the brain without the signal loss typical of exogenous labels (Zhuang et al., 2023).

These studies also revealed an important feature of ferritin reporters. Because ferritin expression is coupled to endogenous iron handling pathways, MRI contrast can be strengthened through coordinated regulation of iron uptake and storage. Strategies combining ferritin with transferrin receptor expression have therefore become a common approach for improving *in vivo* sensitivity.

#### 4.1.2 Immune cell dynamics and the inflammatory microenvironment

In addition to monitoring stem cells, the utility of endogenous iron reporters extends fundamentally into immunology. Because the precise spatiotemporal trafficking of immune populations ultimately dictates disease outcomes, researchers are now deploying ferritin-based systems to continuously map these mobile cells *in vivo*. Rather than relying on static histological snapshots, this genetic labeling strategy captures the live migration and targeted accumulation of macrophages within inflamed or malignant tissues, thereby uncovering the hidden kinetics that drive pathological progression (Ahrens and Bulte, 2013). Macrophages play an important role in tissue repair and chronic inflammation and possess a strong capacity for iron handling, making them attractive targets for ferritin-based imaging strategies. Early studies demonstrated that ferritin overexpression enables non-invasive MRI visualization of macrophage homing and accumulation at sites of inflammation or within tumors (Lee et al., 2017). More recent work has extended this capability to monitor macrophage infiltration in cancer microenvironments, where tumor-associated macrophages contribute to immune suppression and disease progression (Leftin et al., 2019). Ferritin reporters have revealed the spatial distribution and persistence of these cells with high anatomical detail, enabling spatial assessment of macrophage accumulation in relation to tumor progression and therapeutic outcome.

Beyond passive tracking, ferritin-based systems are beginning to capture functional states of immune cells. By linking reporter expression to inflammation-responsive promoters, investigators can capture the temporal dynamics of microglia activation in the central nervous system or macrophage polarization in peripheral tissues. These approaches provide not only localization but also information about inflammatory activity, offering a non-invasive window into disease progression. For example, in models of neuroinflammation and autoimmune disorders, ferritin reporters have highlighted regions of active immune cell recruitment, revealing patterns of immune-cell recruitment that are difficult to characterize using conventional histological approaches alone (DeRosa and Leftin, 2021; Khodanovich et al., 2020).

These studies illustrated the expanding role of ferritin-based MRI reporters in immunological research. Their ability to visualize immune-cell recruitment and monitor inflammatory activity over time has provided new opportunities to study disease progression *in vivo*. As reporter designs become increasingly responsive to cellular function, ferritin-based systems may offer a useful framework for linking anatomical imaging with immune biology.

### 4.2 Monitoring transgene expression and gene therapy

The initial validation of ferritin as a reliable biological surrogate for gene expression was predominantly established within solid tumor models. Early investigators engineered dual-reporter cassettes—coupling optical luminescence with iron-storage capabilities—to elegantly confirm that the geographical boundaries of the MRI signal faithfully perfectly mirrored actual transgene activation *in vivo* (Ono et al., 2009). Crucially, these foundational models also illuminated the environmental dependency of the reporter mechanism. Research utilizing nasopharyngeal carcinoma (NPC) xenografts demonstrated that the intensity of the MRI readout is not exclusively governed by the sheer volume of ferritin overexpression. Instead, the signal can be deliberately amplified and fine-tuned through systemic iron supplementation, establishing a powerful strategy to overcome low inherent sensitivity in iron-depleted microenvironments (Feng et al., 2012).

Moving beyond localized tumor visualization, the most profound application of this technology lies in resolving a major bottleneck in translational medicine: the longitudinal tracking of gene therapy. Clinically, validating a therapeutic payload remains robustly active over extended timeframes typically necessitates highly invasive serial biopsies. Iron-storage systems offer an elegant circumvention. Early hepatic models revealed that chronic H-ferritin overexpression in the liver maintains stable, detectable contrast even as the organ endures continuous dietary iron fluctuations and age-related metabolic shifts (Ziv et al., 2010).

Recently, this remarkable biological compatibility has been successfully translated into practical virology. When researchers delivered iron-storage cassettes via recombinant AAV (rAAV) vectors into mouse livers, the resulting hypointense MRI signals exhibited strict dose-dependency. More importantly, this contrast persisted uninterrupted for upwards of 6 months, accurately reporting the functional status of the vector despite the continuous turnover of host hepatocytes (Moha et al., 2026). Through these progressive advancements, ferritin has evolved from an experimental imaging construct into a highly durable, biopsy-free surveillance platform for evaluating genetic interventions.

### 4.3 *In Vivo* applications in disease Modeling

Iron-storage protein-based MRI reporter systems have evolved from simple cell-labeling tools into versatile platforms for interrogating complex biological processes *in vivo*. Their ability to generate stable, endogenous contrast enables longitudinal tracking of cellular fate, while integration with regulatory elements allows functional readouts of disease-associated dynamics. In each case, the reporters do more than simply mark cell location—they reveal dynamic biological processes, providing insights that were previously inaccessible by non-invasive means.

#### 4.3.1 Tumor imaging and detection of malignant transformation

Initial oncologic applications of ferritin reporters relied heavily on constitutive expression to delineate the physical boundaries of expanding malignancies. As early as 2005, H-ferritin expression in C6 glioma tumors was shown to increase cellular iron content and transverse relaxation rates, producing detectable hypointense contrast *in vivo* without exogenous contrast-agent administration (Cohen et al., 2005). This established ferritin as an endogenous MRI reporter for tumor gene-expression imaging.

However, conventional ferritin labeling can identify the location of transplanted cells, but it cannot distinguish untransformed cells from cells that have undergone malignant transformation. To address this critical safety blind spot, contemporary reporter architectures have evolved from constitutive labeling to surveillance. By deliberately placing the FTH1 cassette under the control of regulatory elements such as hTERT and hypoxia-responsive elements, the MRI signal remains metabolically silent in healthy grafts but is vigorously activated upon tumorigenic conversion (Yang Y. et al., 2016).

A compelling demonstration of this “smart” sentinel strategy utilized the PEG3 promoter to drive FTH1 expression in glioma models. Rather than waiting for a macroscopic mass to form, this engineered construct generated pronounced T2* contrast strictly within the transformed cellular populations—providing an unequivocal MRI readout weeks before the malignancy could be detected via conventional imaging modalities (Sun et al., 2021). Ultimately, this genetic wiring transforms the MRI reporter from a cell tracker into a highly sensitive, early-warning diagnostic platform for cellular therapies, ensuring that subtle oncogenic shifts are captured in real time.

#### 4.3.2 Neurological applications: cell migration, integration, and functional neurogenesis

The mammalian central nervous system (CNS) presents formidable anatomical imaging barriers. Because the opaque cranium and blood-brain barrier severely restrict optical modalities, MRI remains uniquely positioned to interrogate deep neural circuits. The potential of endogenous iron reporters in neurobiology was elegantly established by Iordanova and Ahrens, who tracked native neuroblast migration along the rostral migratory stream. This milestone demonstrated that genetically encoded contrast could faithfully map the continuous spatiotemporal trafficking of endogenous cell populations deep within the intact brain (Iordanova and Ahrens, 2012).

Translating this concept to regenerative medicine, investigators have progressively relied on ferritin constructs to decode the biological fate of neural stem and progenitor cell (NSPC) grafts. Ferritin-labeled NSPC have been tracked following transplantation, enabling longitudinal assessment of graft survival and migration within the host brain (Vande Velde et al., 2012). Building on these demonstrations, Bright Ferritin-based reporters further improved the sensitivity of cell tracking. Neural progenitor cells expressing Bright Ferritin remained detectable for several months after transplantation, allowing visualization of migration toward the quantitative estimation of viable grafted cells over time (Luo et al., 2024).

Most importantly, when coupled with advanced promoter engineering, these systems bridge the gap between anatomical mapping and functional neurogenesis. Building upon the NSE-driven strategies discussed earlier (He et al., 2023), reporter activation can be strictly synchronized with neuronal maturation. In hostile microenvironments like the injured spinal cord, this means the MRI contrast exclusively deepens as therapeutic grafts commit to mature neural phenotypes. Collectively, these studies demonstrate that iron-storage reporters can provide information beyond cell localization, linking MRI contrast to biological processes such as neuronal maturation and graft incorporation into host circuitry. This capability is particularly valuable in regenerative neurology, where understanding the long term behavior of transplanted cells remains a central challenge.

#### 4.3.3 Cardiovascular regeneration: monitoring graft survival in the infarcted heart

The infarcted myocardium presents a profoundly hostile microenvironment that decimates the vast majority of transplanted cells. Consequently, evaluating the efficacy of emerging cardiovascular cellular therapies strictly requires continuous, long term monitoring of graft retention. Historically, tracking these transplants relied on pre-labeling cells with exogenous superparamagnetic iron oxide nanoparticles (SPIONs) (Mathiasen and Kastrup, 2013; Korchinski et al., 2015). However, this approach suffers from a critical translational flaw: upon graft death, the inert SPIONs are aggressively phagocytosed by infiltrating host macrophages, generating persistent false-positive MRI signals that mask the true viability of the therapeutic cells.

To circumvent this deceptive background, the cardiovascular field pivoted toward genetically encoded iron-storage reporters. Comparative studies definitively established that, unlike passive nanoparticle loading, endogenous ferritin expression tightly correlates with active cellular viability (Naumova et al., 2014; Naumova et al., 2010). A pivotal translational leap occurred when Campan and colleagues successfully tracked ferritin-expressing stem cells in a rat model of myocardial infarction using a standard clinical 1.5-T MRI scanner, thereby proving that genetically encoded contrast could be detected under clinically relevant imaging conditions (Campan et al., 2011).

Building upon, contemporary cardiovascular applications have achieved remarkable precision. For instance, recent studies utilizing optimized Bright Ferritin constructs have successfully monitored human pluripotent stem cell-derived cardiomyocytes (hPSC-CMs) following transplantation into infarcted rat hearts over an unprecedented 12-week timeframe (Zhuang et al., 2026). Because the engineered contrast is strictly dependent on the survival and active metabolism of the transplanted cardiomyocytes, researchers could leverage on-demand signal recall to definitively distinguish viable therapeutic grafts from the necrotic scar tissue. This capability secures direct *in vivo* evidence that engineered cardiomyocytes can endure the post-infarct environment and functionally integrate into the host myocardium.

Ultimately, the progression of these diverse preclinical models highlights a fundamental paradigm shift in molecular imaging. While genetic iron-storage reporters are not the absolute gold standard for every imaging scenario, they have indisputably matured into a fundamentally important assessment tool for advanced therapies. By transitioning from inert contrast agents to responsive, living biosensors, this technology provides the essential spatiotemporal and functional readouts necessary to drive the next-generation of cellular and genetic interventions.

## 5 Challenges and limitations

Although iron-storage MRI reporters have made remarkable progress, several fundamental challenges must still be addressed before they can transition from preclinical promise to routine clinical use. Moving these systems from controlled murine models to human applications requires overcoming the inherent boundaries of magnetic resonance physics, resolving the metabolic toll of manipulated iron homeostasis, and navigating rigorous translational safety standards.

### 5.1 Challenges in detection and quantitative interpretation

The moderate sensitivity of ferritin-based contrast remains a primary limitation. MRI contrast arises indirectly from perturbations in local magnetic susceptibility rather than signal amplification, and substantial intracellular iron accumulation is required to produce detectable contrast, often necessitating expression levels or exogenous iron supplementation. Quoted detection thresholds need care, because a cell number is uninterpretable without the voxel volume, field strength and sequence to which it applies. The figure of 10^4^–10^5^ cells that is commonly cited derives from clinical cell-tracking experience rather than from a per-voxel physical limit: in the first clinical MRI cell-tracking study, ferumoxide-labelled dendritic cells carrying approximately 30 pg Fe per cell were detected at 3 T at a resolution of 0.5 × 0.5 × 3.5 mm, with sensitivity estimated by parallel ^111^In-oxine scintigraphy at about 1.5 × 10^4^ cells (Bulte, 2009). Transferred to preclinical imaging the figure is not merely conservative but impossible: a 100 µm isotropic voxel has a volume of 1 nL and can physically accommodate only of the order of 10^3^ mammalian cells, so a requirement of 10^4^ cells per voxel could never be satisfied at that resolution. The comparison that readers require is with exogenous labelling under matched conditions. Single cells labelled with micron-sized iron oxide particles have been detected *in vivo* (Shapiro et al., 2006), and iron oxide labelling routinely delivers 5–30 pg Fe per cell, whereas ferritin and transferrin receptor overexpression in mesenchymal stem cells yielded approximately 0.3–1 pg Fe per cell even with iron supplementation (Pereira et al., 2015). This ten-to hundred-fold deficit in iron per cell, compounded by the far lower relaxivity per iron atom of ferrihydrite compared with magnetite (Section 2.5), accounts for most of the sensitivity gap between genetically encoded and exogenously labelled cells, and it is the reason early engraftment events remain difficult to visualise. And detecting these probes is fundamentally complicated by their negative contrast profile. Within endogenous iron pools like the hepatic microenvironment, faint reporter signals are rapidly drowned out. More critically, while positive emission tools (like PET or CEST) yield highly distinct readouts, iron-induced hyperintensities suffer from diagnostic ambiguity. The specificity of ferritin-derived contrast remains a concern, as similar signal reductions may also occur in tissues where susceptibility effects originate from causes unrelated to reporter activity, like bleeding (Si et al., 2024). While manganese-dependent variants such as Bright Ferritin shift the readout to positive contrast and thereby avoid part of this ambiguity, pushing this technology to the sub-millimeter precision demanded by clinical oncology still necessitates highly customized pulse sequences and advanced post-processing algorithms (Vande Velde et al., 2011).

Closely related to detection sensitivity is the challenge of quantitative interpretation. In contrast to PET, for which signal intensity can often be related directly to tracer concentration, the MRI signal produced by iron-storage reporters reflects a more complex relationship between reporter expression and image contrast. Changes in ferritin levels influence MRI readouts indirectly through their effects on intracellular iron handling and local magnetic susceptibility, making signal intensity dependent on both biological context and imaging conditions. As a result, identical levels of reporter expression may not necessarily produce comparable MRI contrast across different tissues or experimental settings. This complexity is reflected in experimental studies showing that increases in ferritin expression are not always accompanied by proportional changes in transverse relaxation rates (Strbak et al., 2021). Therefore, dark voxels on a scan cannot be mathematically translated into absolute cell counts. This non-linear relationship becomes acutely problematic within diseased tissues, which are inherently chaotic microenvironments. When a host experiences localized vascular damage or actively recruits iron-scavenging immune cells to a lesion, these natural pathophysiological events spontaneously concentrate endogenous iron. As a result, the host tissue generates its own competing signal voids. Disentangling the engineered reporter contrast from this turbulent biological background currently stands as the most profound barrier to achieving true quantitative precision.

### 5.2 Biological consequences of iron perturbation

Beyond technical limitations, perturbation of iron homeostasis introduces important biological constraints. Iron is tightly regulated because it supports a wide range of cellular functions while simultaneously possessing the capacity to drive oxidative damage when present in excess. Disruption of this balance, particularly through forced ferritin overexpression, may alter cellular behavior in unintended ways. For instance, experimental models have shown that overexpression of ferritin heavy chain can affect cell proliferation and migration, suggesting that reporter systems may not be entirely biologically inert (Arosio and Levi, 2010). Similarly, combined overexpression of ferritin and transferrin receptor has been shown to modulate endogenous iron regulatory pathways, leading to compensatory changes in iron metabolism and gene expression (Pereira et al., 2015; Mehta, 2023). These findings raise important concerns regarding the interpretation of imaging data, as the reporter itself may influence the biological processes under investigation.

Safety considerations are further compounded by the potential for iron-induced oxidative stress. Although ferritin sequesters iron in a relatively inert ferrihydrite form, excess intracellular iron can still contribute to reactive oxygen species generation under certain conditions, particularly when iron supplementation strategies are employed to enhance MRI contrast (Galaris et al., 2019; Galy et al., 2024). These effects are often not immediately apparent in short-term experiments but become relevant when cells must survive and function for months or years *in vivo*. These observations suggest that reporter expression and iron supplementation cannot be optimized solely for imaging performance, but must also be evaluated in the context of long term cellular function.

A separate and more specific safety question attaches to the manganese-dependent reporters, and it has not so far been addressed in this literature. Bright Ferritin generates contrast only after systemic MnCl_2_ administration, and because the signal is recalled at each imaging session the exposure is repeated for as long as the cells are followed. Reported subcutaneous doses are 0.4 mmol/kg in mice and 0.2 mmol/kg in rats (Zhuang et al., 2023; Luo et al., 2024), approximately 40- to 80-fold above the 5 μmol/kg used clinically with mangafodipir, the only manganese-based contrast agent to have entered routine practice. Manganese has a narrow window in manganese-enhanced MRI: intravenous LD_50_ values for MnCl_2_ are approximately 93 mg/kg in rats and 38 mg/kg in mice, and tolerance depends strongly on concentration, infusion rate and route of administration (Silva et al., 2004). Cardiac toxicity is dose-dependent and mechanistically specific, with calcium transient amplitude and L-type calcium current both reduced at 50–100 µM MnCl_2_ in isolated hearts (Lamonzie et al., 2022); consistent with this, transient impairment of calcium handling and contractility, reversible on withdrawal of manganese, was observed *in vitro* in the cardiomyocyte tracking study (Zhuang et al., 2026). In humans, chronic manganese exposure produces an extrapyramidal syndrome accompanied by basal ganglia deposition visible as T1 hyperintensity in the globus pallidus (Khindri and Maj, 2025). None of this precludes the approach, and no overt toxicity has been reported at the doses used preclinically. It does mean, however, that the manganese-dependent reporters exchange the probe-free character of the T2/T2* systems for repeated administration of a neurotoxic metal, and that establishing a tolerable cumulative dose over a tracking study lasting months is a prerequisite for translation rather than a detail to be settled afterwards.

### 5.3 Barriers to clinical translation

Although humanized ferritin variants reduce immunogenicity, any residual non-human sequences or bacterial components (e.g., MagA) may still trigger immune responses in immunocompetent hosts (Fu et al., 2010; Yang C. et al., 2016). The clinical translation of genetically encoded MRI reporters is subject to stringent regulatory requirements that extend beyond those applied to conventional imaging agents. Regulatory approval will require evidence that reporter constructs do not produce unacceptable off-target effects and that vector delivery can be performed safely under GMP-compliant manufacturing conditions. Particular scrutiny is likely to focus on the long term consequences of integrating vectors (Wang et al., 2019; Zhang et al., 2023; Doudna and Charpentier, 2014). In addition, the need for specialized MRI sequences and quantitative iron mapping adds complexity to clinical work and increases the technical burden compared with simpler PET-based approaches.

The delivery vehicle carries risks of its own, which are now well documented and deserve to be stated rather than alluded to. Neutralizing antibodies against AAV capsids are common in the general population, with seropositivity of 58%–75% reported for the serotypes most used clinically and prevalence rising with age, which constrains both eligibility and any prospect of redosing (Ertl, 2022; Chhabra et al., 2024). Capsid-directed T cell responses can eliminate transduced cells and terminate expression. Serious adverse events, including complement activation with thrombotic microangiopathy, hepatotoxicity and dorsal root ganglion pathology, occur predominantly at high systemic vector doses (Ertl, 2022), and in one dose-escalation trial of systemic AAV8 gene therapy four participants died of cholestatic liver failure (Shieh et al., 2023). Two considerations apply with particular force to reporter genes. First, dorsal root ganglion pathology in non-human primates has been most pronounced with high-dose reporter transgenes rather than therapeutic ones, which is a direct concern for a construct whose only function is to generate signal. Second, an overexpressed reporter protein is itself a potential source of antigen: in primates followed for more than 2 years, immunogenic transgenes were associated with greater loss of both vector DNA and expression than non-immunogenic ones (Greig et al., 2024). That same study found expression from episomal genomes declining over the first 90 days before stabilising, with more than 10% of hepatocytes retaining vector DNA that no longer produced transcript, and vector integration detectable in approximately 1 in 100 cells (Greig et al., 2024). Durable reporter expression may therefore depend on the integrated rather than the episomal fraction, which is precisely the fraction whose long-term safety is hardest to establish.

These factors highlight that, while iron-storage MRI reporters offer a powerful platform for noninvasive imaging, substantial methodological and translational hurdles must be addressed before their routine clinical adoption. Despite these limitations, continued progress in reporter development and a better understanding of iron handling *in vivo* are likely to improve both safety and imaging performance before clinical translation can be considered. With thoughtful engineering and systematic validation, iron-storage MRI reporters have a realistic path toward becoming safe, effective tools in precision medicine.

## 6 Comparative analysis with emerging CEST and multimodal reporter systems

The value of iron-storage platforms is best judged against the alternatives. Two are relevant here: chemical exchange saturation transfer (CEST) reporters, which generate contrast without metal ions, and multimodal systems that pair an MRI reporter with a PET or optical readout (Gilad and Shapiro, 2017; Bricco et al., 2023). Each addresses a different physical limitation of iron-based contrast, and the comparison indicates where the approaches are complementary rather than competing.

Reporter genes of CEST represent a different paradigm of MRI contrast generation. Instead of relying on iron accumulation to produce negative T2/T2* contrast, CEST reporters exploit the exchange of saturated protons between biomolecules and bulk water to generate contrast without any metal ions (Ward and Balaban, 2000). Prototypical systems such as lysine-rich proteins (LRPs) and their redesigned variants (e.g., rdLRP) enable “multi-color” MRI by encoding distinct resonance frequencies, allowing simultaneous imaging of multiple biological processes within the same tissue (Gilad et al., 2007). Importantly, CEST contrast can be directly linked to protein concentration, offering improved potential for quantitative imaging. However, realizing this potential requires navigating severe physical constraints. Since the contrast relies on proton exchange, the readout is highly vulnerable to localized shifts in tissue acidity or thermal fluctuations, and is frequently obscured by the dense macromolecular environment *in vivo* (Mensah et al., 2025). And from a hardware perspective, saturating these exchangeable protons demands sustained radiofrequency irradiation. This not only elevates specific absorption rates (SAR) to potentially prohibitive levels but also imposes a strict reliance on pristine magnetic field homogeneity—conditions that are notoriously difficult to maintain in deep anatomical structure (Perlman et al., 2020; Gilad et al., 2023).

In parallel, multimodal reporter systems have been developed to integrate MRI with highly sensitive imaging modalities such as PET. These platforms typically combine MRI reporters with PET tracers or fluorescent proteins, enabling validation of signal localization and quantitative assessment of gene expression. For example, the human-derived NIS/OATP1B3 dual system combines the anatomical resolution and soft tissue contrast of MRI with the exquisite sensitivity and quantitative power of PET. This system achieves high signal-to-noise ratios and can detect even sparse transgene expression, while the human-origin proteins minimize immunogenicity (Shalaby et al., 2022). But they often require the administration of exogenous probes and involve increased experimental complexity, limiting their practicality for longitudinal studies. Moreover, differences in spatial resolution and signal origin across modalities can introduce challenges in data integration and interpretation. Another shortcoming is the need for radioactive tracers and the associated radiation dose, as well as the logistical complexity of dual-modality imaging suites—factors that may restrict widespread clinical adoption compared with a purely MRI approach.

While the iron-storage reporters may lack the straightforward quantitative linearity of CEST and the detection thresholds of PET, they offer a combination of autonomous signal generation and long-term stability. The T2/T2* iron reporters avoid both the radiofrequency power deposition of CEST and the radiotracer dependency of PET, which suits them to prolonged *in vivo* surveillance such as tracking stem cell engraftment or confirming the persistence of gene therapy. This particular advantage belongs to the iron-based systems alone: the manganese-dependent reporters require a systemically administered metal at every imaging session and in this respect resemble PET more closely than a probe-free method, notwithstanding that the agent is not radioactive. Recognizing that physical trade-offs are inevitable, the vanguard of the field is now moving beyond competition toward architectural hybridization. By engineering chimeric constructs—such as tethering ferritin cores to CEST domains or integrating human PET modules into a single transcript (Vande Velde et al., 2013)—future reporter systems will likely fuse the autonomous stability of iron with the precision of these complementary modalities, forging a new frontier in high resolution biological sensing.

## 7 Translational potential and clinical roadmap

The clinical translation of iron-storage protein-based MRI reporter genes will depend less on further proof that these systems can generate contrast in animals than on whether they can be integrated safely and reproducibly into clinic. Realizing this potential requires dismantling a complex triad of barriers encompassing (i) vector delivery, (ii) systemic safety, and (iii)radiological standardization.

The most immediate translational bottleneck lies in systemic delivery. Recombinant adeno-associated virus (rAAV) vectors have emerged as leading candidates due to their favorable safety profile, sustained transgene expression, and increasing clinical adoption across multiple therapeutic indications (Wang et al., 2019). Recent clinical successes of AAV mediated gene therapies show the feasibility of stable gene expression in humans, thereby providing a realistic method for integrating MRI reporter genes into therapeutic constructs. Beyond the classic challenges of optimizing tissue tropism, investigators must navigate the host’s immune landscape. Pre-existing neutralizing antibodies against viral capsids, combined with the potential for T cell mediated rejection of cells expressing high levels of exogenous reporters, pose severe risks to long term signal stability (Mingozzi and High, 2013; Muhuri et al., 2021). Thus, a major challenge will be maintaining durable transgene expression in the presence of host immune responses, an issue that remains the focus of ongoing efforts in vector development. The convergence of reporter gene imaging with therapeutic gene delivery presents unique opportunities for real-time monitoring of treatment efficacy. Such theranostic approaches align closely with the principles of precision medicine, where treatment decisions are informed by dynamic biological information.

Equally critical is the need for long term safety evaluation. Reporter gene systems involve persistent or semi-permanent genetic modification, necessitating comprehensive assessment of potential influence to cellular physiology. Therefore, securing regulatory approval demands far more than basic efficacy data. Investigative teams must rigorously map the entire *in vivo* lifecycle of the reporter construct—proving unequivocally that the genetic payload does not inadvertently accumulate in off-target organs or compromise long term host DNA integrity. Parallel to this stringent biological scrutiny, advancing to human cohorts strictly dictates the establishment of clinical manufacturing infrastructure (Naldini, 2015). These requirements reflect a fundamental shift from pharmacological imaging paradigms toward gene based diagnostics, with correspondingly higher thresholds for safety and reproducibility.

From an imaging perspective, translation also depends on the standardization and quantification of MRI. Variability between scanners can substantially influence contrast and complicates cross-centre comparison. Part of this variability has a straightforward physical basis: because the transverse relaxation rate of ferritin rises approximately linearly with field strength (Section 2.5), contrast measured at 7–9.4 T in rodents will be roughly two-to six-fold weaker at the 1.5–3 T available clinically, and cannot be extrapolated without explicit correction for field strength and sequence. Recent advances in quantitative MRI methodologies, particularly relaxometry and quantitative susceptibility mapping (QSM), provide more reproducible and biologically interpretable measurements than conventional weighted imaging, thereby offering a promising work for the quantitative assessment of reporter gene expression (Nagar et al., 2025; Musetta et al., 2024; Ning et al., 2024; Bilgic et al., 2024). Howerver, robust calibration strategies that link imaging readouts to underlying biological parameters remain to be fully established.

Moving forward, the integration of advanced computational tools may help address one of the major barriers to translation. Historically, interpreting iron contrast has relied heavily on human visual assessment of subtle hyperintensities—a practice inherently limited by observer variability and significantly compromised when evaluating complex pathological iron overload (Nasiri et al., 2026). Artificial intelligence and radiomic profiling have been proposed as a route past this subjectivity (Pacheco-Torres, 2026), although we are not aware of any study that has applied either to an iron-storage reporter gene.

The computational foundation for this shift is already being established. Deep learning architectures are currently revolutionizing QSM, recent models have proven highly adept at untangling endogenous iron artifacts and stripping away structural background noise in deep anatomical regions (Yao et al., 2023; Zhou et al., 2025). Algorithms of this kind could in principle be adapted to separate reporter-derived contrast from confounding susceptibility sources, but to our knowledge this has not yet been attempted. Furthermore, as recently validated in targeted cellular imaging, extracting radiomic features allows investigators to objectively decode the spatiotemporal heterogeneity of living cells *in vivo*, transcending the limitations of traditional measurements (Maxouri et al., 2026). It should be emphasised that the studies cited in this paragraph concern quantitative susceptibility mapping and radiomics in other contexts (Yao et al., 2023; Zhou et al., 2025; Maxouri et al., 2026); their extension to reporter-gene imaging is a proposal rather than a demonstrated capability. Coupling genetic engineering to computational quantification is nonetheless the most plausible route by which these reporters could become quantitatively reliable in a clinical setting (Figure 3).

**FIGURE 3:**
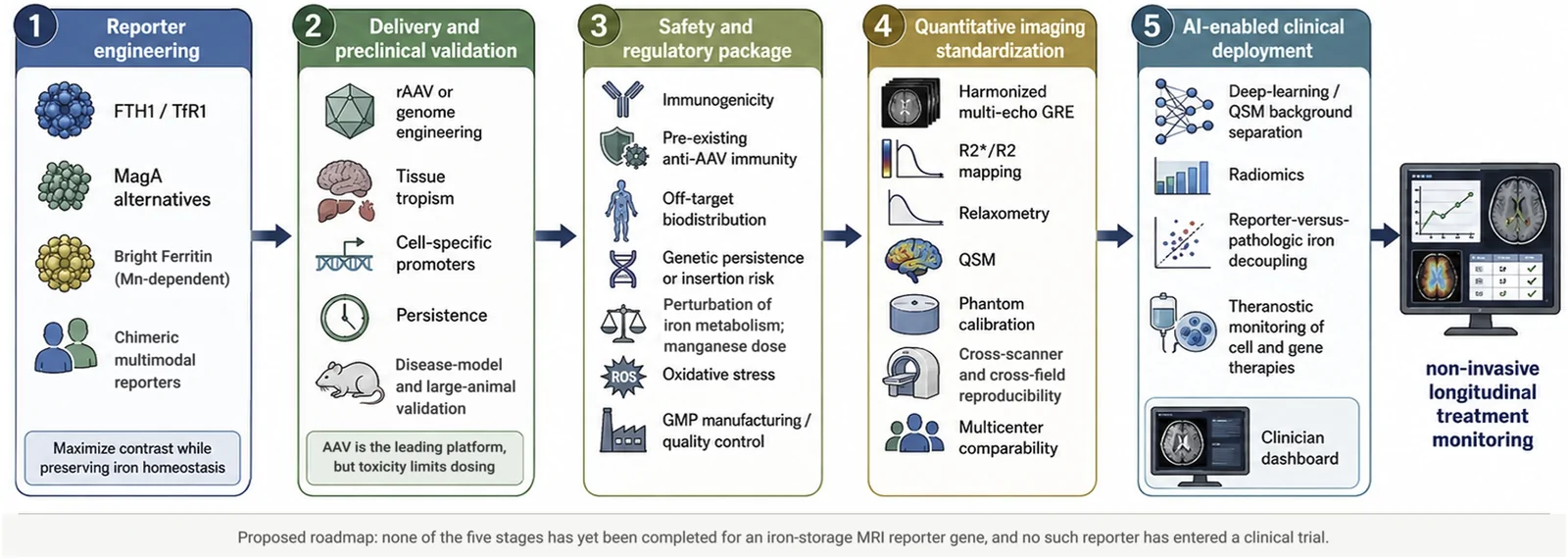
Proposed roadmap toward clinical translation of iron-storage protein-based MRI reporter genes. Successful clinical implementation will require coordinated advances in reporter engineering, vector delivery, safety evaluation, and imaging standardization. Key milestones include development of humanized reporter constructs, optimization of AAV-based delivery systems within the dose range at which vector-related toxicity remains acceptable, comprehensive assessment of long-term biological safety, definition of a tolerable cumulative manganese dose for the manganese-dependent reporters, establishment of GMP-compliant manufacturing pipelines, and validation in large-animal models. Parallel progress in quantitative MRI techniques, including relaxometry and quantitative susceptibility mapping (QSM), together with artificial intelligence-assisted image analysis, may improve signal interpretation and reproducibility. The convergence of reporter-gene technology, precision imaging, and computational analysis is expected to facilitate future applications in regenerative medicine, gene therapy, and personalized healthcare.

## 8 Conclusions and future perspectives

Iron-storage MRI reporters have progressed from a proof of principle to a set of usable experimental tools. Two decades of work have established that a genetically encoded protein can generate MRI contrast that is inherited through cell division, that this contrast can be placed under transcriptional control so that it reports a biological event rather than only a location, and that it can persist for months *in vivo*. For the particular task of following a defined cell population over long intervals without repeated administration of a label, the T2/T2* iron reporters have no close alternative at present.

The limitations are equally clear and, taken together, more restrictive than any individual study suggests. Sensitivity is bounded both by the amount of iron a cell will tolerate and by the low relaxivity per iron atom of ferrihydrite, which leaves reporter-expressing cells one to two orders of magnitude less detectable than cells labelled with iron oxide. Because ferritin relaxation scales with field strength, results obtained at 7–9.4 T do not transfer intact to clinically available field strengths. The contrast is negative and therefore ambiguous against haemorrhage, calcification and endogenous tissue iron, and it is not linearly related to reporter expression, so cell number cannot be recovered from signal intensity. The manganese-dependent variants that circumvent the negative-contrast problem do so at the cost of repeated systemic exposure to a neurotoxic metal at doses well above those used clinically. Delivery still depends on viral vectors whose immunological and genotoxic constraints are now well characterised, and targeted integration mitigates rather than removes them. Almost all of the evidence reviewed here is preclinical: the single demonstration at a clinically available field strength dates from 2011, and no iron-storage reporter has yet entered a clinical trial.

Progress will therefore depend less on further demonstrations that contrast can be generated than on addressing these constraints directly. The most consequential improvement would be a higher relaxivity per expressed protein, since sensitivity is the binding constraint and no refinement of image analysis compensates for a signal that is not present; engineered mineral phases and chimeric constructs are the most plausible route to it. The field would also benefit from reporting conventions that make studies comparable—relaxivity with an explicit normalisation basis, detection limits with the voxel volume, field strength and sequence to which they apply, and metal content per cell—and from head-to-head comparison against exogenous labelling under matched conditions, which is still rarely performed. Quantitative susceptibility mapping and machine-learning reconstruction may eventually help to separate reporter signal from endogenous susceptibility, although neither has yet been applied to this problem. Whether these systems reach the clinic will be decided by whether sensitivity and safety can be improved together, and that question remains open.
